# National Cancer Diagnosis Audits for England 2018 versus 2014: a comparative analysis

**DOI:** 10.3399/BJGP.2022.0268

**Published:** 2023-05-31

**Authors:** Ruth Swann, Sean McPhail, Gary A Abel, Jana Witt, Lorna Wills, Sara Hiom, Georgios Lyratzopoulos, Greg Rubin

**Affiliations:** Cancer Research UK, London.; National Cancer Registration and Analysis Service, NHS Digital, Leeds.; University of Exeter Medical School (Primary Care), University of Exeter, Exeter.; Cystic Fibrosis Trust, London; former NCDA programme manager, Cancer Research UK, London.; Cancer Research UK, London.; NHS Implementation & External Affairs; former director, Cancer Intelligence, Early Diagnosis and Clinical Engagement, Cancer Research UK, London.; University College London, London.; Newcastle University, Newcastle.

**Keywords:** cancer, clinical audit, diagnosis, investigations, morbidity, primary care

## Abstract

**Background:**

Timely diagnosis of cancer in patients who present with symptoms in primary care is a quality-improvement priority.

**Aim:**

To examine possible changes to aspects of the diagnostic process, and its timeliness, before and after publication of the National Institute for Health and Care Excellence's (2015) guidance on the referral of suspected cancer in primary care.

**Design and setting:**

Comparison of findings from population-based clinical audits of cancer diagnosis in general practices in England for patients diagnosed in 2018 or 2014.

**Method:**

GPs in 1878 (2018) and 439 (2014) practices collected primary care information on the diagnostic pathway of cancer patients. Key measures including patient characteristics, place of presentation, number of pre-referral consultations, use of primary care investigations, and referral type were compared between the two audits by descriptive analysis and regression models.

**Results:**

Among 64 489 (2018) and 17 042 (2014) records of a new cancer diagnosis, the percentage of patients with same-day referral (denoted by a primary care interval of 0 days) was higher in 2018 (42.7% versus 37.7%) than in 2014, with similar improvements in median diagnostic interval (36 days versus 40 days). Compared with 2014, in 2018: fewer patients had ≥3 pre-referral consultations (18.8% versus 26.2%); use of primary care investigations increased (47.9% versus 45.4%); urgent cancer referrals increased (54.8% versus 51.8%); emergency referrals decreased (13.4% versus 16.5%); and recorded use of safety netting decreased (40.0% versus 44.4%).

**Conclusion:**

In the 5-year period, including the year when national guidelines were updated (that is, 2015), there were substantial improvements to the diagnostic process of patients who present to general practice in England with symptoms of a subsequently diagnosed cancer.

## INTRODUCTION

Timely diagnosis of cancer is a public and policy concern in many high-income countries worldwide, as longer time to diagnosis is associated with worse clinical and patient-reported outcomes.^1^ Of patients diagnosed with cancer, >90% present with symptoms and most of them consult with a GP in the first instance.^2^^,^^3^ As a consequence, successive UK cancer strategies have included early diagnosis initiatives specifically targeted at primary care, including the creation of urgent referral pathways, together with clinical guidelines supporting their use,^4^ the development of clinical decision support tools,^5^ greater use of safety netting,^6^ and easier access to specialist investigations.^7^ In 2015, guidance from the National Institute for Health and Care Excellence (NICE) on the management and referral of patients with suspected cancer was extensively revised and, for the first time, explicitly specified a 3% risk threshold (for as-yet-undiagnosed cancer) for urgent referral.^8^^,^^9^

Before 2018, two national population-based audits of cancer diagnosis in primary care were undertaken in England; these covered patients diagnosed in 2009–2010^10^ and 2014.^11^ These audits provided unique insights into the diagnostic processes of cancer in primary care, identifying patient groups at greater risk of prolonged diagnostic intervals (defined as the time from first relevant presentation to primary care until diagnosis date) and providing benchmarks by which improvements in patient care could be assessed. In 2019–2020, a third national audit took place, covering patients diagnosed in 2018.

Using the same methodology and, for the most part, questions identical to those used in the 2014 audit, the 2018 audit aimed to characterise the quality of the diagnostic process for patients diagnosed with cancer in 2018, following the revision of NICE guidance in 2015. In this article, the principal findings of the 2018 audit are reported and compared with those from 2014. The authors' hypothesis was that the implementation of NICE guidance should be associated with an observable change in the audited process measures. Data are presented here in a format designed to facilitate a ‘side-by-side’ comparison with the previously reported findings of the 2014 audit.

## METHOD

Data were collected using the same system as the 2014 audit.^11^ Briefly, all incident malignant cancer cases among England residents in 2018^12^ were assigned to the general practice where patients were registered at the time of the diagnosis. Practices were free to choose whether to participate. In 72 of 170 (42.4%) clinical commissioning group (CCG) areas — that is, NHS organisations responsible for the commissioning and planning of healthcare services in a distinct geographically defined population — with participating practices (from a total of 191 CCGs at the beginning of the audit), some form of funding to participate in the National Cancer Diagnosis Audit (NCDA) was offered by the CCG, Cancer Alliance, or other organisation.

**Table:** How this fits in

There is ongoing national monitoring of elements of the cancer-referral process from primary care, including the proportion of urgent referrals (‘2-week waits’) and emergency presentations. The 2014 National Cancer Diagnosis Audit provided a richer picture of this process, as reported by GPs themselves. This research presents a direct comparison of that audit to the more recent one carried out on patients diagnosed in 2018, with revised National Institute for Health and Care Excellence guidance on referral of suspected cancer having been published in 2015. It shows improvements in practice over time such as fewer pre-referral consultations and a shortened time to referral and diagnosis.

Data collection between the 2018 and 2014 audits was similar except for two variables:

- ethnicity — in the 2014 audit, ethnicity was reported by GPs using the 2001 census ethnicity categories. By 2018, ethnicity was complete in cancer registration data for >95% of cases, so this was used to reduce the recording burden on GPs; and
- avoidable delay — in the 2014 audit, GPs were asked about whether an avoidable delay to diagnosis had occurred as a binary variable, and then to detail the nature and subsection of the referral pathway when this occurred. In the 2018 audit, GPs selected from a drop-down menu for each subsection of the referral pathway whether an avoidable delay had occurred. More details on the methods are given in Supplementary Box S1.

### Analysis

Key variables are described by:

- reported gender at diagnosis;
- age group (0–24 years, 25–49 years, 50–64 years, 65–74 years, 75–84 years, and ≥85 years);
- ethnicity (Asian, Black, mixed, other, White, not known);
- deprivation quintile (based on the 2019 Index of Multiple Deprivation [IMD] of the patient's lower super output area of residence); and
- 20 cancer sites to match those reported for the 2014 audit^11^ — namely, bladder, brain, breast, cancer of unknown primary, colon, endometrial, leukaemia, liver, lung, lymphoma, melanoma, multiple myeloma, oesophageal, oral/oropharyngeal, ovarian, pancreatic, prostate, rectal, renal, stomach, and other. All details of the ‘other’ cancer group are listed in Supplementary Table S1.

In line with research guidance,^13^ the authors reported on the following timescales using the definitions given:

- patient interval (PI) — the number of days between symptom onset and the first presentation to a health professional;
- primary care interval (PCI) — the number of days from the first relevant presentation to the date of first referral;
- diagnostic interval (DI) — the number of days from first relevant presentation to the date of diagnosis; and
- the number of days from referral to the date the patient was informed that they had cancer by a specialist.

Interval times of <0 days and >730 days were excluded, consistent with previous literature.^14^ The distribution of gender, age, stage at diagnosis, and cancer site of the NCDA cohort was compared with the 2018 national cancer registration statistics.^15^ Participating and non-participating practices were compared in terms of their key characteristics and key aspects of patients' experience of primary care, similarly to Swann *et al*.^11^

### Regression

To account for potential confounding between other patient-level exposure variables, the relationship between NCDA year, and binary diagnostic process variables was assessed using mixed-effects logistic regression (reference: 2014 audit), with a random effect for general practice. As the continuous diagnostic process variables, pre-referral consultation number, PCI, and DI have a skewed distribution, quantile regression was used as commonly used in relevant literature;^16^^,^^17^ this enabled the authors to understand differences at the median of those variables and for patients at the 90th centile (those who have a large number of pre-referral consultations or long PCI/DI). For the quantile regression model, quality-assurance checks were carried out on the number of pre-referral consultations for cancer-related symptoms in the year up to referral. From the 2018 and 2014 NCDAs, respectively, 21 (0.03%) and 16 (0.09%) tumour-level record entries of >20 consultations were assigned a value of 20, as higher values were deemed to represent implausible clinical scenarios or inputting errors.

Crude and adjusted models are reported. Models were adjusted for gender, age group, ethnicity (Asian, Black, mixed, other, White, and not known), IMD deprivation quintile, and cancer site. Ethnicity data from the National Cancer Registration and Analysis Service (NCRAS) were used, as these were available for both audits. Individuals with ‘not known’ studied diagnostic process measures were excluded from respective analyses. As it was not mandatory to report variables for screening cases in the 2014 audit, the NCRAS-verified screening cases (*n* = 3896) were excluded from the 2018 audit prior to regression analysis so a direct comparison could be made.

In order to examine whether there was selection bias in the included audit cancer cases, the authors carried out a sensitivity analysis of the characteristics of practices that completed <95% versus ≥95% of their NCDA cases. In that analysis, the authors aimed to include a random effect for the practice in order to examine the likely influence of clustering of observations by general practice. Quantile regression models (outcome being interval length) did not converge when including a practice random effect, however, so the sensitivity analysis that included a practice random effect was carried out after dichotomising the PCI and DI (above/below the median and 90th percentile) and using mixed-effects logistic regression models. Analyses were carried out using RStudio (version 2021.09.1+372) and R (version 4.1.2).

## RESULTS

### Sample characteristics

Data were collected on 64 489 cancers diagnosed in 2018 (19.9% of incident cancers in that year).^15^ In total, 1878 practices submitted data (26.2% of English practices), of which 60.5% (*n* = 1137) submitted data for ≥95% of their patients diagnosed with cancer during the audit year. Of the 439 practices that participated in the 2014 NCDA, 226 (51.5%) also participated in the 2018 round. Practice characteristics for the 2018 and 2014 audits are described in Supplementary Table S2 and Supplementary Table S3, respectively; more details are included in Supplementary Box S2.

In the 2018 audit, 22.4% of cases originated from Greater London practices, compared with 12.4% in 2014; this may have reflected the London-wide funding that supported participation in the 2018 audit (Supplementary Table S4).

Supplementary Table S5 and Supplementary Table S6 show the patient characteristics for the 2018 and 2014 audits, respectively. In the 2018 NCDA, 90.6% of the cohort were White (versus 94.7% in the 2014 audit). Directly comparing the distribution of stage at diagnosis between the two cohorts is challenging as the percentage of patients with known stage increased from 76.6% in 2014 to 82.2% in 2018; however, when considering cases with known stage at diagnosis, similar proportions of patients were diagnosed at stages I–II (54.7% in both 2018 and 2014), and stage IV (25.5% and 26.8% in 2018 and 2014, respectively). Stage distributions were also consistent with nationwide stage distributions for the corresponding incident cohorts (that is, 2014 and 2018).^15^ Excluding screening-detected cases in either audit wave produced highly similar findings (data not shown).

Table 1 shows the sample composition of the 2018 audit; this was very similar to that of the 2014 audit (Supplementary Table S7) in terms of key characteristics, for example, proportions in each age group were within 1% of each other. A comparison of the distribution of cancer sites in the 2018 and 2014 audits is shown in Supplementary Figure S1; in total, in 2018, 15.3% of patients were diagnosed with prostate cancer compared with 12.5% in the 2014 audit. The patient cohort in 2018 was similar to the cancer registration data from the same year for age, gender, cancer type, and stage (Supplementary Figure S2).

**Table 1.** Sample composition and referral type^a^ that led most directly to the cancer diagnosis, by demographic characteristic in the 2018 audit (*n* = 64 489)

	**Referral type**										
	**Total, *n* (%)**	**TWW, *n* (%)**	**Urgent, ^b^ *n* (%)**	**Direct access test and upgrade, *n* (%)**	**MDC or RDC, *n* (%)**	**Routine, *n* (%)**	**Screening, ^c^ *n* (%)**	**Emergency, ^d^ *n* (%)**	**To private care, *n* (%)**	**Other, *n* (%)**	**Not known, *n* (%)**
**Total**	64 489 (100.0)	35 366 (54.8)	1786 (2.8)	738 (1.1)	292 (0.5)	3621 (5.6)	4851 (7.5)	8617 (13.4)	875 (1.4)	3704 (5.7)	4639 (7.2)

**Gender**											
**Male**	33 360 (51.7)	19 118 (57.3)	973 (2.9)	379 (1.1)	146 (0.4)	2111 (6.3)	856 (2.6)	4624 (13.9)	500 (1.5)	2069 (6.2)	2584 (7.7)
**Female**	31 129 (48.3)	16 248 (52.2)	813 (2.6)	359 (1.2)	146 (0.5)	1510 (4.9)	3995 (12.8)	3993 (12.8)	375 (1.2)	1635 (5.3)	2055 (6.6)

**Age**											
**0–24 years**	664 (1.0)	192 (28.9)	41 (6.2)	10 (1.5)	7 (1.1)	53 (8.0)	9 (1.4)	228 (34.3)	13 (2.0)	47 (7.1)	64 (9.6)
**25–49 years**	6405 (9.9)	3617 (56.5)	216 (3.4)	60 (0.9)	20 (0.3)	526 (8.2)	457 (7.1)	680 (10.6)	132 (2.1)	298 (4.7)	399 (6.2)
**50–64 years**	16 224 (25.2)	8912 (54.9)	432 (2.7)	160 (1.0)	65 (0.4)	988 (6.1)	2170 (13.4)	1554 (9.6)	285 (1.8)	738 (4.5)	920 (5.7)
**65–74 years**	19 189 (29.8)	10 705 (55.8)	472 (2.5)	252 (1.3)	88 (0.5)	1024 (5.3)	1833 (9.6)	2236 (11.7)	213 (1.1)	1121 (5.8)	1245 (6.5)
**75–84 years**	15 310 (23.7)	8681 (56.7)	431 (2.8)	202 (1.3)	80 (0.5)	786 (5.1)	338 (2.2)	2396 (15.6)	171 (1.1)	999 (6.5)	1226 (8.0)
**≥85 years**	6697 (10.4)	3259 (48.7)	194 (2.9)	54 (0.8)	32 (0.5)	244 (3.6)	44 (0.7)	1523 (22.7)	61 (0.9)	501 (7.5)	785 (11.7)

**Ethnicity ^e^**											
**White**	55 618 (86.2)	30 591 (55.0)	1550 (2.8)	655 (1.2)	253 (0.5)	3084 (5.5)	4069 (7.3)	7591 (13.6)	645 (1.2)	3255 (5.9)	3925 (7.1)
**Asian**	2374 (3.7)	1274 (53.7)	67 (2.8)	21 (0.9)	11 (0.5)	127 (5.3)	213 (9.0)	294 (12.4)	20 (0.8)	135 (5.7)	212 (8.9)
**Black**	1877 (2.9)	1082 (57.6)	55 (2.9)	14 (0.7)	10 (0.5)	132 (7.0)	123 (6.6)	215 (11.5)	7 (0.4)	86 (4.6)	153 (8.2)
**Mixed**	320 (0.5)	167 (52.2)	7 (2.2)	4 (1.3)	1 (0.3)	18 (5.6)	36 (11.3)	39 (12.2)	3 (0.9)	18 (5.6)	27 (8.4)
**Other**	1241 (1.9)	644 (51.9)	44 (3.5)	21 (1.7)	10 (0.8)	63 (5.1)	106 (8.5)	151 (12.2)	27 (2.2)	66 (5.3)	109 (8.8)
**Not known**	3059 (4.7)	1608 (52.6)	63 (2.1)	23 (0.8)	7 (0.2)	197 (6.4)	304 (9.9)	327 (10.7)	173 (5.7)	144 (4.7)	213 (7.0)

**IMD quintile**											
**1 – least deprivation**	13 049 (20.2)	7310 (56.0)	343 (2.6)	121 (0.9)	45 (0.3)	747 (5.7)	1128 (8.6)	1448 (11.1)	409 (3.1)	668 (5.1)	830 (6.4)
**2**	12 736 (19.7)	7236 (56.8)	352 (2.8)	127 (1.0)	46 (0.4)	675 (5.3)	1030 (8.1)	1541 (12.1)	219 (1.7)	681 (5.3)	829 (6.5)
**3**	12 695 (19.7)	7044 (55.5)	357 (2.8)	139 (1.1)	62 (0.5)	742 (5.8)	889 (7.0)	1665 (13.1)	157 (1.2)	737 (5.8)	903 (7.1)
**4**	12 633 (19.6)	6829 (54.1)	345 (2.7)	164 (1.3)	70 (0.6)	703 (5.6)	938 (7.4)	1814 (14.4)	65 (0.5)	739 (5.8)	966 (7.6)
**5 – greatest deprivation**	13 376 (20.7)	6947 (51.9)	389 (2.9)	187 (1.4)	69 (0.5)	754 (5.6)	866 (6.5)	2149 (16.1)	25 (0.2)	879 (6.6)	1111 (8.3)

a *Referral-type information was based on GP inspection of the patient record.*

b *Urgent referrals not for suspected cancer.*

c *Referral type is displayed as entered by the GP, and the screening referral group contains cases that were not confirmed to be screen-detected cases by NCRAS (*n *= 955); the group included patients with breast (*n *= 3277), cervical (*n *= 267), colon (*n *= 500), rectal (*n *= 305), anal (*n *= 5), prostate (*n *= 192), lung (*n *= 82), and ‘other’ (*n *= 223) cancers. All GP and NCRAS-verified screening cases have ‘screening’ as the referral type (*n *= 3896) and comprised patients with breast (*n *= 3042), cervical (*n *= 168), colon (*n *= 419), rectal (*n *= 265), and anal (*n *= 2) cancers.*

d *Emergency referral includes instances of patient self-referral.*

e *NCRAS ethnicity data were used for the 2018 audit. The screening cases have known ethnicity categories, which are presented here. In Supplementary Table S5, ethnicity for NCRAS-verified screening cases is described separately as a direct comparison with the 2014 audit,^11^ in which the GP-reported ethnicity categories were not known for these cases. IMD = Index of Multiple Deprivation. MDC = multidisciplinary centre. NCRAS = National Cancer Registration and Analysis Service. RDC = rapid diagnostic centre. TWW = 2-week wait (urgent referral for suspicion of cancer).*

### Consultations, investigations, and referrals

Table 2 shows that 47.9% of patients had at least one primary care-led investigation. This percentage was 45.4% in the 2014 audit (Supplementary Table S8 and Table S9), and the proportion was greater in males in both audits (2018 audit: 59.4% male versus 35.5% female; 2014 audit: 56.3% male versus 34.3% female). Of investigations carried out, there was a slight increase in blood-test use (37.5% versus 34.6%) but use of imaging (19.8% versus 19.6%) remained constant across the two audit rounds. Cancer biomarker testing in males increased in 2018 (27.3% compared with 22.2% in 2014), which was predominately in prostate cancer (76.2% in 2018 and 70.6% in 2014). In females, cancer biomarker testing was used in 5.4% and 4.2% of females in 2018 and 2014, respectively (Supplementary Table S10).

**Table 2.** Primary care-led investigations ordered by the GP as part of the diagnostic assessment prior to referral by gender and cancer site in the 2018 audit (*n* = 62 559)^a^

	**Investigation group, *n* (%)**				**Patients investigated by test type, *n* (%)**				

	**None^b^**	**Any**	**Not known**	**Blood**	**Urinary**	**Imaging**	**Endoscopy**	**Symptomatic FIT**	**Other**
**Total**	32 592 (52.1)	29 967 (47.9)	1930	23 443 (37.5)	856 (1.4)	12 377 (19.8)	849 (1.4)	104 (0.2)	1793 (2.9)

**Gender**									
**Male**	13 132 (40.6)	19 236 (59.4)	992	15 876 (49.0)	605 (1.9)	6444 (19.9)	475 (1.5)	57 (0.2)	1042 (3.2)
**Female**	19 460 (64.5)	10 731 (35.5)	938	7567 (25.1)	251 (0.8)	5933 (19.7)	374 (1.2)	47 (0.2)	751 (2.5)

**Cancer**									
**Bladder**	643 (39.7)	977 (60.3)	50	603 (37.2)	238 (14.7)	215 (13.3)	10 (0.6)	1 (0.1)	253 (15.6)
**Brain**	658 (76.5)	202 (23.5)	28	154 (17.9)	7 (0.8)	83 (9.7)	1 (0.1)	0 (0.0)	13 (1.5)
**Breast**	9208 (96.0)	384 (4.0)	262	245 (2.6)	3 (0.0)	193 (2.0)	5 (0.1)	2 (0.0)	29 (0.3)
**CUP**	582 (47.2)	651 (52.8)	59	515 (41.8)	12 (1.0)	376 (30.5)	15 (1.2)	2 (0.2)	29 (2.4)
**Colon**	2039 (44.9)	2499 (55.1)	115	2297 (50.6)	28 (0.6)	660 (14.5)	155 (3.4)	40 (0.9)	145 (3.2)
**Endometrial**	905 (55.5)	726 (44.5)	36	388 (23.8)	29 (1.8)	467 (28.6)	7 (0.4)	2 (0.1)	90 (5.5)
**Leukaemia**	541 (35.0)	1005 (65.0)	44	972 (62.9)	11 (0.7)	185 (12.0)	6 (0.4)	1 (0.1)	30 (1.9)
**Liver**	475 (48.6)	502 (51.4)	35	425 (43.5)	9 (0.9)	301 (30.8)	18 (1.8)	2 (0.2)	11 (1.1)
**Lung**	3438 (43.5)	4460 (56.5)	233	2238 (28.3)	23 (0.3)	3877 (49.1)	70 (0.9)	6 (0.1)	163 (2.1)
**Lymphoma**	1059 (42.8)	1415 (57.2)	91	1086 (43.9)	11 (0.4)	810 (32.7)	28 (1.1)	2 (0.1)	51 (2.1)
**Melanoma**	2711 (92.8)	210 (7.2)	48	62 (2.1)	0 (0.0)	35 (1.2)	0 (0.0)	1 (0.0)	129 (4.4)
**Multiple myeloma**	299 (31.8)	641 (68.2)	32	590 (62.8)	9 (1.0)	257 (27.3)	11 (1.2)	0 (0.0)	13 (1.4)
**Oesophageal**	748 (51.1)	716 (48.9)	31	576 (39.3)	2 (0.1)	171 (11.7)	152 (10.4)	2 (0.1)	34 (2.3)
**Oral/oropharyngeal**	888 (74.6)	302 (25.4)	51	198 (16.6)	1 (0.1)	180 (15.1)	3 (0.3)	0 (0.0)	22 (1.8)
**Other**	3227 (53.4)	2814 (46.6)	270	1704 (28.2)	70 (1.2)	1823 (30.2)	51 (0.8)	6 (0.1)	211 (3.5)
**Ovarian**	394 (34.9)	734 (65.1)	21	576 (51.1)	12 (1.1)	511 (45.3)	19 (1.7)	2 (0.2)	33 (2.9)
**Pancreatic**	638 (36.1)	1127 (63.9)	55	984 (55.8)	23 (1.3)	560 (31.7)	64 (3.6)	4 (0.2)	51 (2.9)
**Prostate**	1662 (17.4)	7865 (82.6)	312	7632 (80.1)	297 (3.1)	793 (8.3)	20 (0.2)	1 (0.0)	245 (2.6)
**Rectal**	1179 (50.7)	1147 (49.3)	68	1056 (45.4)	5 (0.2)	132 (5.7)	87 (3.7)	23 (1.0)	99 (4.3)
**Renal**	899 (49.2)	930 (50.8)	65	580 (31.7)	64 (3.5)	574 (31.4)	20 (1.1)	4 (0.2)	91 (5.0)
**Stomach**	399 (37.7)	660 (62.3)	24	562 (53.1)	2 (0.2)	174 (16.4)	107 (10.1)	3 (0.3)	51 (4.8)

a *Not known values have been excluded from percentage calculations. This is to prevent under-reporting of the proportion of the known categories by assuming that the not known cases are missing at random and therefore evenly distributed among the known groups.*

b *Screening and not applicable cases are included in the ‘None’ investigations category. CUP = cancer of unknown primary. FIT = faecal immunochemical test.*

Of patients with at least one relevant pre-referral consultation (*n* = 45 732, 70.9%), 81.2% had <3 pre-referral consultations, compared with 73.8% in the 2014 audit (Supplementary Table S11). In 2018, the proportion of patients who had <3 pre-referral consultations was 73.2% for those with primary care-led investigations and 93.7% for those without, compared with 62.4% and 88.8%, respectively, in the 2014 audit.

For 21 811 of 54 551 (40.0%) patients in 2018, there was documented evidence that safety netting had been used; this compares with 6465 of 14 685 (44.0%) patients in 2014 (data not shown).

The proportion of 2-week wait (TWW) referrals increased by 3.0% in 2018, compared with 2014 (54.8% versus 51.8%), while emergency (13.4% versus 16.5%), urgent (2.8% versus 4.4%), and routine (5.6% versus 7.9%) referrals decreased by 3.1%, 1.6%, and 2.3%, respectively; the 2018 data are shown in Table 1.

In the 2018 audit, compared with 2014, there was a lower proportion of emergency referrals in those aged 0–24 years (34.3% versus 47.5%) and >85 years (22.7% versus 29.1%), and for patients with brain cancer (54.8% versus 64.9%), multiple myeloma (17.3% versus 27.9%), and leukaemia (26.1% versus 35.1%) (Supplementary Table S12). The proportion of routine referrals was 5.1% and 5.4% points lower in 2018 than in 2014 for patients with multiple myeloma (9.2% versus 14.3%) and prostate cancer (6.7% versus 12.1%).

### Intervals and avoidable delays

The percentage of patients with same-day referral (denoted by a PCI of 0 days) was higher in 2018 than in 2014 (42.7% versus 37.7%). Overall, median intervals were shorter in the 2018 audit for the PCI (2 days, versus 5 days in 2014) and the DI (36 days, versus 40 days in 2014). Fewer patients in the 2018 audit, compared with the 2014 audit, had a PCI or DI exceeding 60 days and 90 days (PCI >60 days: 9.2% versus 12.5%; PCI >90 days: 5.9% versus 8.3%; DI >60 days: 31.9% versus 35.8%; DI >90 days: 20.8% in 2018 versus 24.0% in 2014) (Supplementary Table S13 [2018 data] and Supplementary Table S14 [2014 data]).

There was large variation in DI by cancer site, consistently, in both audits. The overall decrease in intervals was driven by a shortening of intervals of those cancer sites that had longer-than-average intervals in the 2014 audit, such as multiple myeloma (47 days versus 54 days) or stomach cancer (32 days versus 42 days).

Cases with GP-ordered investigations had longer median PCIs compared with those without (10 days versus 0 days), which was shorter than in the 2014 audit (15 days versus 0 days). For patients who had investigations, compared with those who did not, the DI was longer in the 2018 round (49 days versus 20 days), although this was not as great as the difference observed in the 2014 audit (57 days versus 22 days).

The overall median PI in 2018 (not measured in 2014) was 2 days; however, there was substantial variation by cancer site, with the median PI for oesophageal and rectal cancers approaching 2 weeks. The median interval from referral to the date the patient was informed they had cancer by a specialist was 33 days (interquartile range [IQR] 20–55 days) in the 2018 audit, which was longer than in 2014 (28 days, IQR 16–49 days).

GPs perceived an avoidable delay to a patient's diagnosis in 15 060 (23.4%) cases (Supplementary Table S15). The proportion of patients with a perceived avoidable delay occurring at any one of the pre-presentation, primary care, or post-referral phases ranged from 10.5% to 12.4%. Patients aged 0–24 years had more reported delays between presentation and referral (17.2%), and there was variation by cancer site. For breast cancer, avoidable delay was reported in 9.2% of patients before presentation, and 2.7% and 3.2%, respectively, of patients between presentation and referral, and after referral; however, for patients with rectal cancer, these figures were 20.7%, 14.2%, and 11.9%, respectively. The data on avoidable delays are not directly comparable with the 2014 audit, because of changes in the audit question.

### Direct comparison of 2018 and 2014 audits

Regression modelling confirmed that, after accounting for possible confounding between exposure variables, all observed changes in aspects of the diagnostic process and intervals prevailed (Table 3), except for the median reduction of 3 days in the PCI to 0 days (that is, no change after adjustment). This is a result of zero-inflated distributions causing a lack of resolution at the median, and should be interpreted alongside the increase in the observed proportion of same-day referred patients with a PCI value of 0 days (see above), and the reduction after adjustment at the 90th percentile (observed −19 days, adjusted −16 days).

**Table 3.** Mixed-effects logistic regression to compare the 2018 and 2014 audits for key metrics

	**NCDA 2018, *n* (%)^a^**	**NCDA 2014, *n* (%)^a^**	**Crude OR^b^ (95% CI)**	***P*-value^b^**	**Adjusted OR^c^ (95% CI)**	***P*-value^c^**
**≥3 pre-referral consultations**	8588 (18.8)	3235 (26.2)	0.69 (0.64 to 0.73)	<0.001	0.70 (0.65 to 0.75)	<0.001
**Any investigations**	29 967 (51.1)	7602 (48.2)	1.13 (1.08 to 1.18)	<0.001	1.11 (1.05 to 1.17)	<0.001
**Blood tests**	23 443 (40.0)	5795 (36.8)	1.17 (1.12 to 1.23)	<0.001	1.15 (1.09 to 1.22)	<0.001
**Endoscopies**	849 (1.4)	267 (1.7)	0.83 (0.70 to 0.99)	0.041	0.86 (0.71 to 1.03)	0.103
**Imaging**	12 377 (21.1)	3289 (20.9)	1.01 (0.96 to 1.06)	0.070	1.03 (0.97 to 1.08)	0.3873
**Safety netting**	21 811 (43.1)	6465 (47.3)	0.77 (0.73 to 0.83)	<0.001	0.76 (0.71 to 0.81)	<0.001
**TWW referral**	35 366 (63.2)	8820 (57.7)	1.27 (1.21 to 1.32)	<0.001	1.24 (1.19 to 1.30)	<0.001
**Emergency referral**	8617 (15.4)	2818 (18.4)	0.80 (0.75 to 0.84)	<0.001	0.83 (0.78 to 0.88)	<0.001

a *The percentage of patients in each category differ from those reported elsewhere because of screening cases being removed and only known cases of each category being included in the regression models.*

b *The crude mixed-effects logistic regression had a random effect added for general practice.*

c *The mixed-effects logistic regression was adjusted for gender, age group, ethnicity, IMD deprivation quintile, and cancer site, with a random effect added for general practice. IMD = Index of Multiple Deprivation. NCDA = National Cancer Diagnosis Audit. OR = odds ratio. TWW = 2-week wait.*

In 2018, there was a statistically significant reduction (*P*<0.001) in patients who had ≥3 pre-referral consultations, safety netting, and emergency referrals (odds ratios [ORs] 0.70, 0.76, and 0.83, respectively) and a statistically significant increase (*P*<0.001) in patients who had any investigations, blood tests, or a TWW referral (ORs 1.1, 1.2, and 1.2, respectively) (Table 3). Adjusted quantile regression results showed a median difference of 3 days in the DI in 2018 from 2014. A sensitivity analysis, allowing a random effect for the practice, also provided concordant findings (Supplementary Table S16).

## DISCUSSION

### Summary

For patients diagnosed with cancer in 2018, compared with those diagnosed in 2014, most diagnostic process measures had improved, with notable reductions in PCI length and the proportion of patients experiencing multiple pre-referral consultations. Patients diagnosed in 2018 were statistically significantly more likely to have been referred urgently for suspected cancer (TWW) and less likely to have had an emergency referral compared with those in the 2014 audit. The modest reduction in the DI, compared with the substantial reduction in the length of the PCI, suggests considerable progress in primary care diagnostic processes, not matched by capacity increases in secondary care diagnostic pathways. The apparent increase in the interval between referral and the date the patient was informed of their cancer status should be interpreted cautiously as both audits predate the formal implementation of this measure.

Use of safety netting was advocated in 2015 by the Independent Cancer Taskforce;^6^ however, recorded use of safety netting was lower in 2018 than in 2014. Further discussion points are included in Supplementary Box S3.

### Strengths and limitations

This was a large-scale population-based audit with more than one-quarter of practices in England participating, and cases identified using the English cancer registration system. Detailed information was added from the primary care patient record by practice staff, able to read free-text entries, and able to apply their clinical judgement when completing the audit data fields.

Both audit rounds used similar methods for data collection, enabling — for the most part — between-round comparisons. Further, the characteristics of audited patients and participating practices were highly representative of the national incident cohort in respect of sex, age, and cancer site, and of all English practices in both rounds. There were minor differences in ethnicity case mix reflecting both general demographic trends and the greater participation in the 2018 audit of London practices, and a greater proportion of prostate cancers in 2018 in England.^18^

In spite of similarities in terms of other characteristics, there is still potential for differences in the diagnostic processes between participating and non-participating practices, for example, practices interested in cancer diagnosis and care — which, therefore, may be more likely to be performing well — may be over-represented among participating practices.

In principle, some of the observed changes in the key outcomes of the diagnostic process (the percentage of patients who were referred at first consultation, and the median length of the PCI and the DI) may reflect changes in the distribution of stage at diagnosis between 2014 and 2018. However, answering this question requires causal inferences to be made, which is challenging because of the changing completeness of data on stage at diagnosis, and because the changes in the diagnostic-process outcomes may, themselves, mediate changes in the stage distribution. Nonetheless, as the between-audit waves increase in stage completeness was relatively small, and as the distribution of stage among observed cases was highly similar between the two audits, the findings are consistent with the hypothesis that most of the observed changes in the examined diagnostic outcomes are unrelated to stage distribution and reflect changes in diagnostic processes *per se*.

The reduction in safety netting could reflect fewer patients consulting ≥3 times pre-referral and should be interpreted with caution, as safety-netting advice may be under-recorded.^19^

### Comparison with existing literature

To the authors' knowledge, international experience with population-based audits of the diagnosis of cancer in primary care is limited. Population-based audits have been conducted in sub-national populations of different sizes previously in England, Scotland, and Denmark.^11^^,^^20^^–^^23^ The findings presented here concord with other evidence indicating secular trends in increasing numbers of TWW referrals and a decreasing use of emergency diagnosis.^24^^,^^25^

In comparison with the 2018 Scottish NCDA,^26^ patients in England had fewer multiple consultations (19% versus 25%), more urgent referrals for suspected cancer (55% versus 43%), fewer emergency referrals (13% versus 19%), fewer primary care-led investigations (48% versus 51%), and fewer reported avoidable delays (23% versus 33%).

The 2018 audit also included information on the PI, as recorded in the GP record. This measure was not included in the 2014 audit, but was collected in 2009–2010;^27^ compared with the results of the 2009–2010 audit, there has been a substantial reduction in the measured median PI (from 10 days to 2 days). There are well-recognised difficulties in measuring intervals between symptom onset and presentation (that is, patient intervals) in population studies;^28^ nonetheless, the pattern of variation arising from the two audits is highly similar — oesophageal, rectal, and oropharyngeal cancer had some of the longest PIs, whereas bladder and kidney cancer had some of the shortest.

The diagnostic pathway and routes to diagnosis of patients with lung, colorectal, and ovarian cancer have also been described in bespoke samples of patients in several jurisdictions.^29^^–^^31^ Our findings concord with the overall pattern of variation in diagnostic intervals observed between different cancers, but provide a wider appreciation of key measures of the diagnostic process in a larger population-based sample including all cancer sites, albeit in a single jurisdiction.

### Implications for practice

Variation in PI by cancer site may be useful in directing symptom awareness interventions. The proportion of patients urgently referred with suspected cancer rose from 51.8% to 54.8% between the two audit rounds. Although diagnosis after such a referral is associated with earlier stage and better outcomes than emergency presentation, the continued rise in the use of this pathway may become unsustainable without sufficient uplift in capacity in diagnostic services to meet this rising demand.^32^^,^^33^ Moreover, the optimal referral rate per practice is unknown and health economic data to underpin its provision are lacking.

The use of diagnostic tests in primary care in 2018 had changed very little from 2014, despite the recommendations made by NICE in 2015. Some diagnostic tests (for example, faecal immunochemical tests) were used in only a small percentage of cases. Whether these findings represent under-use of this test, and how their use may have changed since 2018, needs to be further examined.

Regarding the longer PCI and DI for patients who had primary care led-investigations, it should not be assumed that primary care testing necessarily prolongs the diagnostic process — lack of primary care testing, particularly among patients with non-specific symptoms, could have resulted in even longer intervals in the absence of information from these tests. In 2018, there was only a small increase in the proportion of patients with diagnostic tests initiated in primary care, compared with 2014. This was despite the government's commitment, in 2015, to spend an additional £300 million on diagnostic tests by 2020,^34^ which may have been expected to support and encourage more primary care direct access and diagnostics use. Patients with primary care-led investigations had longer PCIs and DIs than those without. It should not be assumed that primary care testing necessarily prolongs the diagnostic process — lack of primary care testing, particularly among patients with non-specific symptoms, could have resulted in even longer intervals in the absence of information from these tests.

The organisation and funding of large-scale audits such as NCDA is challenging, requiring substantial resources for informatics support, as well as general practice engagement and facilitation. The COVID-19 pandemic, which post-dates the findings reported here, has not only had a considerable impact on patients' responses to symptoms of possible cancer, the nature of GP consultations, and referral patterns, but also facilitated the greater sharing of primary care data.^35^ Further audit rounds would be invaluable for the richness of insight they could provide; greater data availability could be leveraged by incorporating elements of newly available data as part of a routine standing audit of cancer diagnosis.

## Data

Data for this audit are based on patient-level information collected by the NHS, as part of the care and support of patients with cancer. The data are collated, maintained, and quality assured by the National Cancer Registration and Analysis Service, which is now part of NHS Digital (it was part of Public Health England during the data-collection phase of the audit). Data for the National Cancer Diagnosis Audit are held by NHS Digital, and can be made available for approved users through the Data Access Request Service: https://digital.nhs.uk/services/data-access-request-service-dars.
